# Onanism in Young Children

**Published:** 1886-12

**Authors:** Professor Hirschsprung

**Affiliations:** Copenhagen


EXPERIENCES CONCERNING ONANISM IN YOUNG CHILDREN. *By* PROFESSOR HIRSCHSPRUNG, *in Copenhagen. Berliner Klinisehe Wochenschrift, September 20, 1886.*

The author of this paper is convinced that masturbation occurs in very young children, though we may be disinclined to believe it; and that the effects on the organism, and especially on the brain, are much worse than after puberty.

"I will show that masturbation is practiced at a very early age, not only by boys but also by girls, most frequently, according to my experience, by the latter.

"The following typical case occurred lately: A shapely, well-nourished girl, just thirteen months old, of Danish parentage, was brought to me on the 20th of November, 1884. She was the only child; the mother seemed very nervous, the father was healthy. The attending physician could not explain the attacks from which the child suffered.

"The child was said to have been suffering for eight or nine months from its attacks, which were still of a doubtful nature.

"On examination absolutely nothing was to be learned. The child was ruddy, was not rickety, had a normal temperature, normal urine, suffered a little from constipation. It cried continually during the investigation, and became quiet for the first time when the nurse took it in her arms. It grew gradually quiet, and I then observed an attack which lasted for about five minutes. The child lay stretched over the breast and shoulder of the girl, clinging to her firmly with its hands, one on the servant's back and the other on her breast. Its feet were braced against the girl's abdomen. Now began a series of to-and-fro motions with the pelvis and the legs which were stretched out parallel. The child worked continually, was perfectly still, red in the face, the pupils dilated, grimaces came and went in its face; it sighed and sobbed so that it was supposed to be in pain, and the girl drew it, pityingly, closer to the breast. During the attack I stood close by. The child watched me with a longing, vacant look—the instant before it could not endure me. When the attack was over it loosened its hands and began immediately to cry again.

"The investigation of the genitals before and after the attack showed nothing specially abnormal.

"Such an attack, according to the mother, occurred several times during the day. By night the child was very restless, it was wakeful and fell asleep for the first time only after it had been taken up and given the opportunity to practice its habit.

"I communicated to my Swedish colleague my opinion of the case, and the advice which, of course, naturally followed from it, to remove from the child the opportunity for its vicious practices.

"After a month, I had the pleasure of learning from the father that my advice had borne fruit, but the family physician could not agree with my interpretation of the facts. Nevertheless, in this case, typical means were used, consisting of friction of the genital organs, the irritation being voluntary, and a high degree of sexual orgasm arose; this means nothing else than an intentional, even though ignorant arousing of the sexual passion. The vice indeed showed itself at an unusually early period of life, but a counterpart occurs in Kraft's case, cited by Vogel, of a girl eleven months old * * * who irritated the vulva with the hand.

"There is the closest agreement in the appearances seen in girls a little older, though the methods vary somewhat. They may be seen going through their exercise bent over a chair, stool, or something of the kind, the genitals pressed against the piece of furniture, but the typical method is crossing the legs while sitting and making backward and forward motions, with flushed face, staring eyes, great anxiety until the culmination. The case often ends with a sigh and collapse. The procedure is repeated whenever the opportunity is offered, often several times a day, perhaps also at night, many times quite typically in sleep.

"In 1884 I had such a girl under observation in the hospital. She was born October 10th, 1881, and was at the time of her reception not yet quite three years old. She belonged to a neurotic family,—father and uncle had committed suicide,—and she was the only child.

"She was in every way well developed, was large for her age, but thin and anæmic, very active, somewhat restless in her manner. Since the age of one and a half years she had had attacks which were explained as pruritus of the genitals. She had visited the 'Poliklinik' of the hospital, but without result, because the supervision on the part of the nervous mother was not stringent enough, therefore she was brought to the hospital. During a considerable portion of the forty days of her stay in the hospital she was kept in bed, with her legs stretched out and bound fast to its sides. She was quite well, but was constipated, and somewhat restless during her sleep. After getting out of bed there were several beginnings of an attack but nothing more."

The author mentions another case of a girl of eight, still under treatment, who has masturbated since her third year. "These cases, with three or four more, in age from one to eight, complete the list. When one can look over such a list he will be more firmly fixed in the opinion which I state positively that the cases occurring earlier in life belong to exactly the same class as the latter."

On account of the attendant erections the diagnosis is easier in males, but such cases are rare, he having seen only three cases in young boys.

" A sixteen months old boy came to the 'Poliklinik' on July 30th, 1878. He was small, thin, with fontanelles still open the head very large; he had twelve teeth and could walk when supported. The mother said that since his eighth month he had been observed daily in the following condition,—of late this had occured oftener: He sits upright, bent a little forward, with his body moving back and forth, penis erect, cries when disturbed, and at last is bathed in a profuse perspiration. After the attack, which may last for half an hour, he falls into a complete stupor. For a month he has been growing thin, has diarrhœa and a poor appetite. Worms have not been noticed. Cold packs were advised. August 2d—Up to this time he has had only two attacks, and these were quickly overcome by cold water.

"There can be no doubt that masturbation in small children does not belong to the things of daily occurrence, but on the other hand, it does not occur so seldom as to escape the observation of a busy pædiatrist, nor must it in all cases be the result of false observation." * * * In works on children the subject is usually only hinted at. "Vogel, I have already stated, mentions the fact, but the most detailed description is in *Steiner's Compendium der Kinderkrankheiten*, 1872. In the chapter on 'Onanism' he says: 'Concerning the age in which this vice is practiced, I have often noticed that it may be detected in very small children—from one to two years of age.' And he here remarks that in nurslings, after weaning, the sucking of the finger, which is so frequent, often occurs along with erection, great redness of the face, increased brightness of the eyes, and, finally, an outburst of perspiration. This remark certainly for the first time gives to it an ætiological meaning and is, according to his opinion, a plain indication of sexual irritation. Steiner does not believe that these occurrences take place in children at the breast." * * *

Dr. Lindner, of Budapest, amplifies the same thought. In a note the author refers to the article by Dr. Jacobi on "Masturbation and hysteria in young children in the American Journal of Obstetrics and Diseases of Women and children, 1876," which he has only seen in abstract. He has himself not seen such cases, but he calls attention to the exact agreement of Steiner and Lindner, and advises the physician to have a watchful eye upon the movements of children while nursing.

"As to my own cases, I will emphasize a distinct impression that I have had that great nervousness, or even decided mental disease, exists in the nearest relatives of these children and doubtless may play a part as a predisposing force.

"In the next place, I have had the impression that relatively many children observed by me have suffered with habitual constipation. I have been still more convinced of the importance of this symptom since my acquaintance with a case which Dr. V. Mohr, of Copenhagen, a few years ago, made the subject of a communication to the medical society of that place.

"He met a girl, three and half years old—again a girl—that had, for some time past, suffered with constipation. Lately she had begun to masturbate. She braced herself with her arms against a table and rubbed the thighs against one another. The mother said that for the whole day and evening, until she went to sleep, she did hardly anything else. Scarcely had she come into Mohr's room when she began her practice. The labia majora and minora were somewhat reddened, no fissure in the anus but a tension on account of which Mohr dilated the anus with his fingers. The operation removed the constipation as well as the masturbation. After eight days the old constipation began again, so that the operation had to be repeated. The child was then for three weeks very bright, then relapsed again, afterwards she recovered, and, at this time, apparently radically.

"I cannot pass over a badly itching attack of urticaria or of lichen, which may be such a great torment of children and is so difficult to remove. These troubles may be the first inducement to handling and friction, and may have onanism as a result."

"I have four times heard ascarides mentioned, but their meaning as a causative force I have not been able to understand." * * *

When Bouchut mentions "*prurit de la vulve* as a frequent cause of onanism I am unable to form an opinion of my own concerning the independent existence of this symptom.

"Instruction by others, which is certainly the most common cause with boys and girls in later years, I may, fortunately, in the first years of life pass over." * * *

"The greatest care must be exercised to discover the cause, and the greatest watchfulness 'early and late, day and night,' must be used to prevent the practice; consequently such patients are usually better treated in hospitals."

LESTER CURTIS.

